# Relevance of fructose intake in adolescence for fatty liver indices in young adulthood

**DOI:** 10.1007/s00394-020-02463-2

**Published:** 2021-01-19

**Authors:** Ines Perrar, Anette E. Buyken, Katharina J. Penczynski, Thomas Remer, Gunter G. Kuhnle, Christian Herder, Michael Roden, Karen Della Corte, Ute Nöthlings, Ute Alexy

**Affiliations:** 1 Institute of Nutritional and Food Sciences , Nutritional Epidemiology, University of Bonn, DONALD Study, Heinstück 11, 44225 Dortmund, Germany; 2 Institute of Nutrition, Consumption and Health, Faculty of Natural Sciences, University Paderborn, Warburger Straße 100, 33098 Paderborn, Germany; 3 Department Food Safety, German Federal Institute for Risk Assessment (BfR), Max-Dohrn-Straße 8-10, 10589 Berlin, Germany; 4 Department of Food and Nutritional Sciences, University of Reading, Whiteknights, RG6 6UR UK; 5 Institute for Clinical Diabetology, German Diabetes Center, Leibniz Center for Diabetes Research at Heinrich Heine University Düsseldorf, Auf’m Hennekamp 65, 40225 Düsseldorf, Germany; 6 German Center for Diabetes Research (DZD), Ingolstädter Landstr. 1, 85764 München-Neuherberg, Germany; 7 Division of Endocrinology and Diabetology, Medical Faculty, Heinrich Heine University Düsseldorf, Moorenstraße 5, 40225 Düsseldorf, Germany; 8 Institute of Nutritional and Food Sciences, Nutritional Epidemiology, University of Bonn, Endenicher Allee 19 B, 53115 Bonn, Germany

**Keywords:** Fructose, Sugar intake, Non-alcoholic fatty liver disease, Adolescence, Young adulthood, Biomarker

## Abstract

**Purpose:**

To examine the association between fructose intake in adolescence and fatty liver indices (hepatic steatosis index (HSI), fatty liver index (FLI)) in young adulthood.

**Methods:**

Overall, 246 participants of the Dortmund Nutritional and Anthropometric Longitudinally Designed (DONALD) study who had a fasting blood sample in adulthood (18–36 years), at least two 3-day weighed dietary records for calculating fructose intakes and other fructose-containing sugars (total (TS), free (FS), added sugar (AS)) as well as two complete 24-h urine samples for calculating sugar excretion (fructose excretion (FE), fructose + sucrose excretion (FE + SE)) in adolescence (males: 9.5–16.5 years; females: 8.5–15.5 years) were analysed using multivariable linear regression analyses.

**Results:**

On the level of dietary intake, no prospective associations were observed between adolescent fructose intake and both adult fatty liver indices, whereas higher FS intakes were associated with lower levels of HSI (*P*_trend_ = 0.02) and FLI (*P*_trend_ = 0.03). On the urinary excretion level, however, a higher FE (P_trend_ = 0.03) and FE + SE (*P*_trend_ = 0.01) in adolescence were prospectively related to higher adult FLI values. No associations were observed between adolescent sugar excretion and adult HSI.

**Conclusion:**

The present study does not provide unambiguous support for a detrimental impact of adolescent fructose intake on adult liver health. Nonetheless, further examinations estimating exposure by means of urinary excretion as well as dietary intake levels appear warranted.

**Supplementary Information:**

The online version contains supplementary material available at 10.1007/s00394-020-02463-2.

## Introduction

Non-alcoholic fatty liver disease (NAFLD) is one of the most common liver diseases worldwide [1] and ranges from simple non-alcoholic fatty liver (NAFL) and non-alcoholic-steatohepatitis (NASH) to fibrosis and cirrhosis [2]. In addition, NAFLD is increasingly recognized as an important risk factor for type 2 diabetes and cardiovascular disease [3]. Among modifiable factors contributing to NAFLD, excessive fructose consumption is regarded a main driver since it may induce de novo lipogenesis, impair fatty acid oxidation [4, 5] and trigger hepatic inflammation [5]. In addition, a high fructose intake is discussed to promote hepatic insulin resistance [5] and very low density lipoprotein (VLDL) synthesis [4]. Indeed, a recent meta-analysis of controlled intervention studies confirmed a short-term detrimental effect of high fructose intakes on hepatic insulin resistance among persons without diabetes, both in studies examining hypercaloric and isocaloric diets [6]. However, evidence on the mid-term and long-term relevance of fructose intake on liver health is scarce [6].

Total sugar (TS), added sugar (AS) and free sugar (FS) include a considerable amount of fructose, e.g. as part of sucrose [7]. Hence, the intake of these sugars may also play a role in the development of NAFLD, by virtue of their metabolic fate, i.e. providing fructose or by contributing glucose, which may also affect liver metabolism, e.g. through hepatic triglyceride synthesis [8]. A randomized clinical trial among male adolescents with NAFLD showed a significant improvement in hepatic steatosis by a diet low in FS [9].

Investigations addressing health consequences of sugar intake may be prone to bias when relying solely on self-reports [10] due to selective under-reporting [11][11]. Therefore, such studies should best be complemented by exposure estimation using validated biomarkers of sugar intake [10, 12]. Urinary fructose (FE) and fructose plus sucrose excretion (FE + SE) are established as predictive biomarkers for sugar intake [10, 12]. Urinary fructose represents dietary fructose as well as fructose from sucrose intake, which escapes the metabolism of liver and other tissues [10]. Urinary sucrose as part of FE + SE is also interesting to investigate since even in a healthy organism, small amounts of dietary sucrose may pass unhydrolyzed through the intestinal wall [13] ending up in the urine [10].

The longer-term consequence of a higher fructose intake during adolescence is of particular interest since this age is regarded as a potentially “critical period” for the development of various diseases in later life [14, 15] and because fructose [16, 17], AS and FS intake levels are high among adolescents [18–20]. Since under-reporting identified based on energy intake levels appears to be particularly problematic during adolescence [21, 22], additional consideration of biomarkers will likely contribute to a more realistic assessment of actual intakes of sugars.

Data on the potential long-term impact of fructose intake in adolescence for liver health in young adulthood have not yet been reported. Therefore, we analysed the prospective association between habitual fructose intake (based on 3-day weighed dietary records in adolescence) as well as fructose excretion as biomarkers for sugar intake (measured in 24-h urine samples) and fatty liver indices (hepatic steatosis index (HSI) and fatty liver index (FLI)) in young adulthood among participants of the DONALD study. In addition, we examined the association between TS, FS and AS intake as well as fructose + sucrose excretion (FE + SE) with fatty liver indices.

## Methods

### DONALD study

The DOrtmund Nutritional and Anthropometric Longitudinally Designed (DONALD) study is an ongoing, open cohort study conducted in Dortmund, Germany, which started to collect information on diet, growth, development and metabolism of healthy children and adolescents in 1985. Since then, 35–40 infants have been newly recruited every year. Eligible participants include healthy German infants (i.e., infants free of diseases affecting growth and/or dietary intake), whose parents are willing to participate in a long-term study and of whom at least one has sufficient knowledge of the German language. The participants are first examined at the age of 3 months and return for three more visits in the first year of life, two in the second year and thereafter annually until young adulthood. Yearly examinations include 3-day weighed dietary records, anthropometric measurements, collection of 24-h urine samples (starting at age 3–4 years), interviews on lifestyle, and medical examinations. Parental examinations (anthropometric measurements, lifestyle interviews) take place every four years. Since 2005, in addition to the regular examinations, participants older than 18 years are invited every five years to provide a fasting blood sample. Further details on the study have been described elsewhere [23, 24]. The study was approved by the Ethics Committee of the University of Bonn according to the guidelines of the Declaration of Helsinki. All examinations are performed with parental and later on, children’s written consent.

### Study sample

At the start of the dataset compilation for the current investigation, 248 participants had provided a fasting blood sample in adulthood (18–36 years) for calculating fatty liver indices as well as at least two 3-day weighed dietary records and two complete 24-h urine samples in adolescence (males: 9.5–16.5 years; females: 8.5–15.5 years) for estimating sugar intake and excretion. Participants further fulfilled the following eligibility criteria: singletons, born at term (37 to < 43 gestation weeks) with normal birth weight.

Two participants had to be excluded from the statistical analyses. Data for fasting blood glucose, needed to identify persons with diabetes for HSI calculation, were not available for one participant. A further participant was excluded because of implausible blood glucose level. The analyses were thus performed with a final number of 246. Per participant, at least two (*n*_records_ = 1,582) dietary records [median (Q1; Q3): 4 (2; 5)] as well as two (*n*_samples_ = 492) 24-h urine samples and one blood sample (*n*_samples_ = 246) were available.

Power calculations indicated that available sample sizes were sufficient to detect associations between dietary intakes during adolescence and adult outcomes with a power of > 80%, using partial correlations of 0.8–0.21.

### Dietary assessment

Dietary data were assessed using 3-day weighed dietary records on three consecutive days. All foods and beverages consumed by the child, as well as leftovers, were weighed and recorded by the parents or by the older participants themselves, with the use of electronic food scales (± 1 g). The participants chose the day of the beginning of dietary recording within a given period of time. When exact weighing was not possible, household measures are allowed for semi-quantitative recording. Information on recipes (ingredients and preparation) and on the types and brands of food items consumed was also requested. Medication and dietary supplement use were also recorded but were excluded from this analysis. A trained dietitian checked the dietary records for accuracy and completeness. Subsequently, energy and nutrition intakes were calculated using our continuously updated in-house nutrient database LEBTAB [25]. The composition of staple foods is based on the German food composition tables BLS 3.02. Energy and nutrient contents of commercial food products, i.e., processed foods and ready-to-eat-meals were estimated by recipe simulation using labelled ingredients and nutrient contents. Within LEBTAB, total fructose amounts did not include that portion of fructose found in the disaccharide sucrose, therefore calculations of TS, FS and AS intake were made which considered both fructose and sucrose. AS in LEBTAB is defined as all sugars that were added to foods during processing or home preparation according to the definition in Cummings & Stephen [26]. Since FS is not included in LEBTAB, the individual intake was calculated for the present investigation. FS was defined according to the definition by the Scientific Advisory Committee on Nutrition (SACN) [27], which includes AS plus sugars from fruit juices, vegetable juices, juice spritzers and smoothies. Energy, nutrient, alcohol and food group intakes as well as dietary glycemic index (GI) were calculated as individual means from all available dietary records during adolescence to describe the participants’ habitual intake.

### Anthropometric measurements

Height and weight are measured by nurses according to standard procedures with the participants dressed in underwear only and barefoot. From the age of 2 years onwards, standing height is measured to the nearest 0.1 cm using a digital stadiometer (Harpenden, Crymych, UK). Body weight is measured to the nearest 100 g using an electronic scale (Seca 753E; Seca Weighing and Measuring System). Waist circumference was measured at the midpoint between lower rib and iliac crest to the nearest 0.1 cm. Triceps and subscapular skinfolds were measured on the right side of the body using a skinfold calliper (Holtain Ltd, Croswell, Dyfed, UK). The sum of both skinfolds was used for the estimation of percentage body fat according to the equations of Slaughter [28] for adolescents and the Durnin–Womersley [29] equations among adults and related to the square of height to obtain fat mass index (FMI, kg/m^2^). Body mass index (BMI, kg/m^2^) was calculated as the body weight (kg) divided by the square of the body height (m). Body surface area (BSA) was calculated according to DuBois and DuBois [30]. Data on height measurements from the age of 6 years onwards in boys and on height measurements from the age of 5 years onwards in girls were used to estimate the pubertal marker age at take-off (ATO) [31] using the parametric Preece & Baines model 1 [32]. Details of the method have been described elsewhere [31]. Maternal body weight and height are measured with the same equipment as for the participants. Maternal overweight was defined as a BMI ≥ 25 kg/m^2^.

### Urine collection and analyses

Annual 24-h urine collections were scheduled in participants older than 3 or 4 years. Collections follow a standardized procedure after detailed instruction of the families. The participants were asked to void their bladders upon getting up in the morning; this micturition was completely discarded. This sets the start of the collection which ends with voiding the bladder the next morning. During the collection period at home, the participants stored the micturitions in preservative-free, Extran-cleaned (Extran, MA03; Merck, Darmstadt, Germany) 1-L plastic containers at less than − 12 °C. After the transfer to the study institute by a dietitian, they were stored at − 22 °C until thawed.

Urinary creatinine and urea excretion were measured in the DONALD laboratory. 24-h creatinine excretion was measured by a creatinine analyser (Beckman-2; Beckman Instruments, Fullerton, CA, USA) using the kinetic Jaffe´ [33] procedure. 24-h urea excretion was determined by Urease-Berthelot method [34, 35].

Urinary fructose and sucrose were measured in the laboratory of the Department of Food & Nutritional Sciences at the University of Reading using LC–MS and quantified using stable-isotope labelled internal standards (^13^C_12_-sucrose and ^13^C_6_-fructose, Sigma Aldrich, Gillingham, UK). After shipping on dry ice, urine samples were stored at − 80 °C until analysis and thawed at 4 °C. An aliquot of 100 µL of urine was combined with 100 µL acetonitrile containing the internal standards, vortex-mixed, centrifuged at 13000* g* for 10 min and the supernatant transferred into a 96-well plate for LC–MS/MS analysis. Samples were separated by HPLC (Acquity BEH Amide 2.1 × 50 mm, 1.7 µm column (Waters, Milford, MA, USA), kept at 35 °C), using 80/20 (v/v) acetonitrile/water with 0.2% NH_4_OH as mobile phase (250 µL/min) using an Acquity UPLC binary solvent manager, sampler manager and column manager (Waters, Milford, MA, USA), and detected by tandem mass spectrometry using a Quattro Ultima tandem quadrupole mass spectrometer (Micromass, Manchester, UK). The mass spectrometer was operated with electrospray ionisation (ESI) in positive ion mode in multiple reaction monitoring (MRM) mode. Nitrogen was used as the desolvation gas and argon was used as the collision gas. The following generic source conditions were used: capillary voltage, 3.6 kV; cone voltage, 35 V; desolvation temperature, 400 °C; source temperature, 120 °C, desolvation gas flow, 500 L/hr; cone gas flow, 100 L/hr. The concentration range was 0.1–500 µmol/L (Fructose: 0.02–90.1 mg/L; sucrose: 0.03–171.2 mg/L). To calculate daily excretions, concentrations were converted to mg/d using the molar mass of fructose or sucrose and multiplied with the 24-h urine volume.

### Blood analyses

Venous blood samples were drawn after an overnight fast, centrifuged at 4 °C within 15 min and stored at − 80 °C in the DONALD study Center. Eleven participants had provided two blood samples until blood analyses in 2015, of which the latest sample was chosen, if the volume of the first sample was not sufficient for all blood analyses. Fasting plasma glucose was determined on a Roche/Hitachi Cobas c 311 analyzer (Basel, Switzerland). Plasma activities of the liver enzymes, alanine-aminotransferase (ALT), aspartate-aminotransferase (AST), gamma-glutamyltransferase (GGT), and plasma concentrations of triglycerides (TG) were measured using a Roche/Hitachi Cobas c311 analyzer (Roche Diagnostics, Mannheim, Germany) at the German Diabetes Center (DDZ) as described [36].

As surrogates for NAFLD, we used fatty liver indices, which have been validated against imaging methods and used in large studies before [37, 38]. Indices of hepatic steatosis were calculated as follows:

Hepatic steatosis index (HSI):

$${\text{HSI}} = 8 \times {{{\text{ALT}}} \mathord{\left/ {\vphantom {{{\text{ALT}}} {{\text{AST}}}}} \right. \kern-\nulldelimiterspace} {{\text{AST}}}} + {\text{BMI}}{.}$$

(+ 2, if female; + 2, if diabetes mellitus, but not applied, as there were no persons with diabetes among our participants) [39],

Fatty liver index (FLI):

$${\text{FLI}} = {{e^{x} } \mathord{\left/ {\vphantom {{e^{x} } {\left( {1 + e^{x} } \right)}}} \right. \kern-\nulldelimiterspace} {\left( {1 + e^{x} } \right)}} \times 100.$$

with $$x\, = \,0.{953}\, \times \,{\text{ln}}\left( {{\text{TG}}} \right)\, + \,0.{139}\, \times \,{\text{BMI}}\, + \,0.{718}\, \times \,{\text{ln}}\left( {{\text{GGT}}} \right)\, + \,0.0{53}\, \times \,{\text{waist circumference}}\, - \,{15}.{745}$$ [40]

### Assessment of further covariates

Further covariates were collected on the child’s admission to the DONALD study or periodically at follow-up visits. The child’s birth characteristics were retrieved from a German standardized pregnancy document called “Mutterpass”. High maternal educational status (≥ 12 years of schooling), maternal employment as well as participant’s smoking status and physical activity in adulthood were inquired with standardized questionnaires. The standardized questionnaire regarding physical activity includes among others questions (e.g. what kind of sport, frequency and duration) on organised (e.g. club sport, gym) and non-organised sports (e.g. playing football with friends, cycling) as well as how the participants get to school (e.g. by car/bus or by feet). The questionnare based on Adolescent Physical Activity Recall Questionnaires [41] and questions from the the German Health Interview and Examination Survey for Children and Adolescents (KiGGS) [42].

### Statistical analyses

The statistical analyses of the present evaluation were performed using SAS® procedures (version 9.2; Cary, NC, USA). The significance level was set at *p* < 0.05. Characteristics in the tables are presented as medians with their interquartile ranges for continuous variables or as frequencies and percentages for categorical variables. Results from the regression analyses are presented as adjusted least-square means (95% CI) by sex-specific tertiles of the respective predictor with *p*-trend values from models with the predictors as continuous variables.

To achieve normal distribution, outcome variables (HSI, FLI) were transformed prior to analysis using log transformations. Since sugar excretions were validated log-transformed as biomarkers for total sugar intake [12, 43], urinary predictor variables (FE, FE + SE) were also transformed. Before calculating the individual means from available records during adolescence, sugar intake (fructose, TS, FS and AS) variables as well as energy-yielding covariables were energy-adjusted by the residual method [44], studentized by age (years) and sex to account for age- and sex-dependent intake differences. Analogously, sugar excretion (fructose, sucrose and the sum of both) was adjusted for BSA (studentized by age group and sex), since it is closely related with individual body size-dependent glomerular filtration rate [45]. Since dietary GI as well as flavonoid intake correlated with fructose intake, these variables were also adjusted for fructose intake using the residual method to avoid multicollinearity in the models. Furthermore, we tested the interaction between the predictor-outcome associations and sex, by which no interactions were found. Prospective associations between sugar intake (fructose, TS, FS and AS) or sugar excretion (FE and FE + SE) during adolescence and fatty liver indices (HSI, FLI) in early adulthood were analysed by multivariable linear regression models, using the transformed variables as explained above. The final models were tested for linearity, heteroskedasticity, multicollinearity and normal distribution.

Crude models (model A) included the exposure variable as well as sex and age at blood withdrawal in adulthood in both analyses (dietary and biomarker). Adjusted models (dietary analyses: models B and C; biomarker analyses: model B) were constructed by individual examination of potential influencing covariates and hierarchical inclusion [46] of those which substantially modified the exposure’s regression coefficient by ≥ 10% [47] or independently predicted the outcome variable [48]. Potential confounding covariates considered for inclusion in the different hierarchical levels were:

1. early life factors [gestational weight gain (kg), maternal age at birth (year), gestational age (week), birth weight (g), first-born child (yes/no), full breastfeeding ≥ 4 months (yes/no)],
2. socioeconomic factors [smokers in the household (yes/no), maternal high educational status (≥ 12 years (yes/no)), maternal employment (yes/no) maternal overweight ( yes/no)],
3. adolescent anthropometrics [mean FMI from available data in adolescence (kg^2^/m)],
  For dietary analyses in addition:
4. dietary variables [dietary GI (residuals, adjusted for fructose intake), dietary fiber intake (energy-adjusted residuals), total flavonoid intake (energy- and fructose intake-adjusted residuals); saturated fatty acids intake (SFA; energy-adjusted residuals), carbohydrate intake (others than sugars; energy-adjusted residuals), ratio plant/animal protein]

For biomarker analyses in addition:

(4) urinary variables [24-h creatinine excretion (mmol/d), 24-h urea excretion (mmol/d), urine volume (L/d)].

For missing values, the respective median of the total sample was used (*n* = 6 for gestational weight gain, *n* = 1 for full breastfeeding ≥ 4 months, *n* = 2 for maternal overweight, *n* = 1 smokers in the household).

Model building was done for the primary exposures, i.e. fructose intake as well as fructose excretion in their relation to both fatty liver indices. To allow comparability of the results, the obtained models were then also used for analyses of the secondary exposures, i.e. TS, FS and AS intake as well as FE + SE in relation to the fatty liver indices.

To use 24-h sugar excretion as a predictive biomarker for sugar intake, Tasevska et al. generated an equation [49] that “calibrates the biomarker to provide an unbiased measure of intake” [10]. As this equation was created among adults (40–69 years), it is not suitable for younger populations, such as children and adolescents. Nevertheless, we performed sensitivity analyses using "calibrated" sugar excretions (calibrated FE, calibrated SE, calibrated FE + SE).

As mentioned in the introduction, adolescents are susceptible of under-reporting energy intake. Records were considered as under-reported when the total energy intake (TEI) was inadequate in relation to the estimated basal metabolic rate (BMR) according to age- and sex-specific equations of Schofield [50], using pediatric cutoffs from Sichert-Hellert et al. [22]. Underreporters were not excluded from the analyses, as this procedure only identifies under-reported energy intake, but no selective under-reporting of food groups [51] or sugar intake [11]. Instead, 167 (10.6%) records, in which intake levels were under-reported, collected by 85 participants, were excluded for sensitivity analyses, i.e. sensitivity analyses were based on 1415 records from 245 participants.

Additional sensitivity analyses in subsamples of participants who had provided the following data were performed in both dietary and urinary models: (a) ATO (*n* = 202), (b) adult smoking status (yes/no, *n* = 224), (c) levels of adult physical activity (low/medium/high; *n* = 169), (d) adult alcohol consumption (g/d, *n* = 195). These variables were considered as potentially confounding factors in regression models of the subsamples, respectively.

## Results

The median follow-up time between the mean age during adolescence and adulthood was 8.6 years in the dietary sample and 8.4 years in the urinary sample (Tables 1, 2). The socioeconomic factors and lifestyle parameters indicate a high socioeconomic status (SES) of the participants of the DONALD study. Sex-specific tertiles of fructose, TS, FS and AS intakes as well as urinary FE and FE + SE are shown in Tables 3 and 4.

**Table 1:** Baseline characteristics of the DONALD participants in adolescence (males: 9.5–16.5 years; females: 8.5–15.5 years): anthropometry, dietary and urinary data as well as early life and socioeconomic factors

	*n*_participants_	*n*_samples_	Dietary sample	Urinary sample
Females/males [%]	246	1582/492	50.8/49.2	50.8/49.2
Age [years]	246	1582/492	12.6 (12.0; 13.0)	12.5 (11.5; 13.5)
Dietary data				
TEI [kcal/d]	246	1582	1949 (1668; 2183)	
Fat [%E]	246	1582	34.9 (31.8; 37.0)	
SFA [%E]	246	1582	15.8 (14.1; 17.3)	
Protein [%E]	246	1582	12.9 (12.1; 14.1)	
Animal/plant protein ratio	246	1582	1.7 (1.4; 2.0)	
Carbohydrate [%E]	246	1582	51.1 (48.4; 54.3)	
Total sugar [%E]	246	1582	26.7 (23.5; 30.7)	
Free sugar [%E]	246	1582	17.6 (14.8; 21.2)	
Added sugar [%E]	246	1582	14.3 (10.7; 16.7)	
Fructose [%E]	246	1582	3.8 (2.8; 4.9)	
Fiber [g/MJ]	246	1582	8.9 (7.7; 10.2)	
Dietary GI	246	1582	56.4 (54.8; 57.8)	
Urinary data				
Fructose excretion [mg/d]	223	446		22.3 (14.2; 32.3)
Sucrose excretion [mg/d]	246	492		27.1 (17.2; 40.5)
Fructose+sucrose excretion [mg/d]	223	446		50.4 (36.7; 72.5)
Creatinine excretion [mmol/d]	246	492		7.6 (6.5; 9.1)
Urea excretion [mmol/d]	246	492		266 (222; 325)
Urine Volume [L/d]	246	492		1.0 (0.7; 1.2)
Anthropometric data				
BMI [kg/m^2^]^c^	246	1582/492	18.9 (16.9; 20.7)	18.1 (16.2; 19.9)
FMI [kg/m^2^]	246	1582/492	3.3 (2.5; 5.0)	3.2 (2.3; 4.5)
Body surface area [m^2^]^a^	246	1582/492	1.5 (1.4; 1.6)	1.4 (1.3; 1.6)
ATO [years]^b^	202	1339/404	9.7 (8.8; 10.5)	9.7 (8.8; 10.5)
Early life factors				
Maternal age at birth [year]	246	1582/492	30 (28; 33)	30 (28; 33)
Birth weight [g]	246	1582/492	3475 (3130; 3820)	3475 (3130; 3820)
Gestational age [week]	246	1582/492	40 (39; 41)	40 (39; 41)
Gestational weight gain [kg]	240	1545/480	12 (10; 15)	12 (10; 15)
Full breastfeeding ≥ 4 months [%]	245	1575/490	47.4	47.4
First child of the family [%]	246	1582/492	61.4	61.4
Socioeconomic factors				
Maternal overweight [%]^c^	244	1568/488	37.3	37.7
Maternal high educational status [%]^d^	246	1582/492	52.0	52.4
Maternal employment [%]	246	1582/492	55.7	59.8
Smokers in household [%]	245	1576/490	29.8	29.8

Values are frequencies (%) or medians (25th; 75th percentile)

*BMI* body mass index, *FMI* fat mass index, *ATO* age at take-off, *TEI* total energy intake, *%E* percentage of total energy intake, *SFA* saturated fatty acid, *GI* Glycemic index

^a^Body surface area according to the equation of DuBois & DuBois [30]

^b^Age at take of according to Buyken et al. [31] using the parametric Preece and Baines model 1 [32]

^c^BMI > 25 kg/m^2^

^d^≥ 12 years of schooling

**Table 2:** Characteristics of the DONALD participants at follow-up in early adulthood (18–36 years): blood, anthropometry, dietary and lifestyle data

	*n*_participants/samples_	
Adult age [years]	246	21.0 (18.1; 24.0)
Blood data		
Glucose [mg/dL]	246	95.1 (90.3; 101.0)
TG [mg/dL]	246	92.0 (71.0; 122.0)
ALT [U/L]	246	15.9 (12.9; 20.9)
AST [U/L]	246	21.1 (18.1; 21.0)
GGT [U/L]	246	14.2 (10.9; 19.0)
HSI	246	29.9 (27.8; 33.5)
FLI	246	8.1 (4.7; 19.1)
Anthropometric data		
BMI [kg/m^2^]	246	22.5 (20.8; 25.1)
FMI [kg/m^2^]	246	5.8 (4.0; 7.3)
Dietary data		
TEI [kcal/d]	195	2149 (1805; 2563)
Fat [%E]	195	33.4 (29.2; 37.9)
SFA [%E]	195	14.9 (12.5; 17.2)
Protein [%E]	195	13.8 (12.5; 15.7)
Animal /plant protein ratio	195	1.7 (1.3; 2.2)
Carbohydrate [%E]	195	49.3 (44.7; 53.9)
Total sugar [%E]	195	22.5 (18.8; 28.7)
Free sugar [%E]	195	14.5 (10.0; 20.0)
Added sugar [%E]	195	11.1 (7.2; 15.5)
Fructose [%E]	195	3.0 (1.7; 4.6)
Fiber [g/MJ]	195	8.6 (7.3; 10.7)
Lifestyle data		
Current smoker [%]	224	27.4
Physical activity (low/moderat/high) [%]	170	32.9/34.2/32.9

Values are medians (25th; 75th percentile) or frequencies (%)

*TG* Triglycerides, *ALT* aminotransferase, *AST* aspartate-aminotransferase, *GGT* gamma-glutamyltransferase, *HSI* hepatic steatosis index, *FLI* fatty liver index, *BMI* body mass index, *FMI* fat mass index, *TEI* total energy intake, *%E* percentage of total energy intake, *SFA* saturated fatty acid

**Table 3:** Tertiles of sugar (fructose, total, free and added sugar) intake

	Low intake (T1)	Moderate intake (T2)	High intake (T3)
Fructose (%E)	2.5 (2.0; 2.8)	3.8 (3.5; 4.2)	5.7 (4.9; 6.8)
Fructose (g/d)	11.8 (8.7; 14.0)	18.6 (15.0; 22.0)	27.1 (22.5; 34.7)
Total sugar (%E)	22.4 (20.0; 23.8)	26.6 (25.6; 27.9)	32.0 (30.7; 33.4)
Total sugar (g/d)	102.9 (87.3; 122.8)	127.6 (106.0; 149.7)	153.6 (136.6; 185.0)
Free sugar (%E)	13.2 (11.1; 15.0)	17.6 (16.6; 18.9)	23.0 (21.2; 24.4)
Free sugar (g/d)	65.5 (47.7; 78.3)	82.3 (70.0; 97.8)	113.1 (98.4; 133.1)
Added sugar(%E)	9.4 (8.1; 10.8)	14.2 (12.4; 14.9)	18.3 (16.7; 20.4)
Added sugar (g/d)	45.8 (34.7; 57.4)	67.4 (54.8; 77.0)	89.6 (74.9; 114.4)

Values are medians (25th; 75th percentile) of intake in the respective sex-specific tertile

*%E* percentage of total energy intake

**Table 4:** Tertiles of urinary sugar excretion (fructose (FE), fructose + sucrose (FE + SE))

	Low excretion (T1)	Moderate excretion (T2)	High excretion (T3)
FE (mg/d)	11.2 (9.3; 14.5)	21.6 (18.8; 26.6)	37.2 (30.5; 54.5)
FE+SE (mg/d)	31.5 (24.0; 37.0)	50.0 (43.3; 56.6)	86.2 (66.7; 122.6)

Values are medians (25th; 75th percentile) of excretion in the respective sex-specific tertile

**Table 5:** Relation of fructose intake as well as total, free and added sugar intake in adolescence (males: 9.5–16.5 years; females: 8.5–15.5 years) with indices of non-alcoholic fatty liver diseases (hepatic steatosis index (HSI), fatty liver index (FLI)) in young adulthood (18–36 years) (n = 246)

	Predicted means for HSI in adulthood in tertiles of sugar intake in adolescence			*P*_trend_	Predicted means for FLI in adulthood in tertiles of sugar intake in adolescence			*P*_trend_
	Low intake (T1)	Moderate intake (T2)	High intake (T3)		Low intake (T1)	Moderate intake (T2)	High intake (T3)	
Fructose intake								
Model A	31.5 (30.5; 32.6)	30.5 (29.5; 31.5)	30.3 (29.3; 31.4)	0.09	10.8 (8.8; 13.3)	9.6 (7.9; 11.7)	9.2 (7.5; 11.2)	0.36
Model B	31.1 (30.2; 32.1)	30.9 (30.0; 31.8)	30.4 (29.5; 31.3)	0.16	10.3 (8.7; 12.6)	10.5 (8.7; 12.6)	9.4 (7.7; 11.3)	0.59
Model C	31.2 (30.3; 32.3)	30.9 (30.0; 31.5)	30.3 (29.3; 31.3)	0.09	10.7 (8.9; 13.0)	10.5 (8.8; 12.6)	8.8 (7.2; 10.7)	0.18
Total sugar intake								
Model A	32.0 (31.0; 33.1)	30.4 (29.4; 31.4)	30.0 (29.0; 31.0)	**0.03**	11.6 (9.4; 14.1)	9.1 (7.4; 11.0)	9.1 (7.4; 11.1)	0.22
Model B	32.0 (31.1; 33.0)	30.3 (29.5; 31.3)	30.3 (29.4; 31.3)	0.05	11.5 (9.5; 13.8)	9.1 (7.6; 10.9)	9.8 (8.1; 11.7)	0.41
Model C	32.3 (31.3; 33.4)	30.4 (29.6; 31.4)	30.0 (29.1; 31.0)	**0.02**	12.4 (10.2; 15.1)	9.2 (7.7; 11.0)	8.9 (7.3; 10.8)	0.07
Free Sugar intake								
Model A	32.3 (31.2; 33.4)	29.7 (28.7; 30.7)	30.4 (29.4; 31.5)	**0.02**	12.8 (10.5; 15.6)	8.0 (6.5; 9.6)	9.4 (7.7; 11.5)	0.09
Model B	32.0 (31.0; 33.0)	30.1 (29.1; 31.0)	30.7 (29.8; 31.6)	**0.04**	12.0 (10.0; 14.4)	8.3 (6.9; 9.9)	9.7 (8.1; 11.6)	0.20
Model C	32.0 (31.1; 33.1)	30.0 (29. 1; 30.9)	30.4 (29.5; 31.4)	**0.02**	12.8 (10.6; 15.5)	8.6 (7.2; 10.3)	9.2 (7.6; 11.1)	**0.03**
Added Sugar intake								
Model A	31.7 (30.7; 32.8)	30.1 (29.1; 31.1)	30.6 (29.6; 31.6)	0.42	11.3 (9.2; 13.8)	8.5 (7.0; 10.4)	9.9 (8.1; 12.1)	0.64
Model B	31.8 (30.8; 32.8)	30.5 (29.5; 31.4)	30.6 (29.7; 31.5)	0.30	11.2 (9.3; 13.6)	9.1 (7.6; 10.9)	10.0 (8.3;12.0)	0.60
Model C	31.8 (30.8; 32.8)	30.5 (29.6; 31.4)	30.5 (29.6; 31.5)	0.25	11.4 (9.4; 13.8)	9.3 (7.7; 11.1)	9.6 (8.0; 11.6)	0.37

Values are least square means (95% confidence interval) of HSI/FLI in the respective sex-specific tertiles of sugar intake

*p *values for models are based on linear multivariable regression analyses (significant *p* values of the models are marked bold). Outcome variables (HSI and FLI) were log-transformed

Model A (crude model) adjusted for sex and age at blood withdrawal

Model B = Model A additionally adjusted for: gestational weight gain (kg), maternal high educational status (yes/no), maternal overweight (yes/no), FMI in adolescence (kg/m2)

Model C = Model B additionally adjusted for energy-yielding nutrients (adolescent saturated fatty acids intake (residuals))

*HIS* hepatic steatosis index, *FLI* fatty liver index

**Table 6:** Relation of fructose excretion (FE) as well as fructose + sucrose (FE + SE) excretion in adolescence (males: 9.5–16.5 years; females: 8.5–15.5 years) with indices of non-alcoholic fatty liver diseases (hepatic steatosis index (HSI), fatty liver index (FLI)) in young adulthood (18–36 years) (*n* = 223)

	Predicted means for HSI in adulthood in tertiles of sugar excretion in adolescence			*P*_trend_	Predicted means for FLI in adulthood in tertiles of sugar excretion in adolescence			*P*_trend_
	Low excretion (T1)	Moderate excretion (T2)	High excretion (T3)		Low excretion (T1)	Moderate excretion (T2)	High excretion (T3)	
FE								
Model A	30.8 (29.7; 31.8)	30.4 (29.4; 31.5)	30.4 (29.4; 31.5)	0.45	9.4 (7.7; 11.5)	9.4 (7.7; 11.6)	9.5 (7.8; 11.7)	0.30
Model B	30.3 (29.4; 31.3)	30.4 (29.5; 31.4)	30.8 (29.9; 31.8)	0.05	8.7 (7.3; 10.4)	9.5 (8.0; 11.4)	10.2 (8.5; 12.1)	**0.03**
FE+SE								
Model A	30.8 (29.7; 31.8)	30.3 (29.2; 31.3)	30.6 (29.6; 31.7)	0.81	9.6 (7.8; 11.7)	8.9 (7.3; 10.9)	9.9 (8.1; 12.1)	0.31
Model B	30.2 (29.3; 31.2)	30.1 (29.2; 31.1)	31.2 (30.3; 32.2)	0.14	8.7 (7.2; 10.4)	8.8 (7.4; 10.5)	11.1 (9.2; 13.2)	**0.01**

Values are least square means (95% confidence interval) of HSI/FLI in respective sex-specific tertiles of sugar excretion

*p* values for models are based on linear multivariable regression analyses (significant *p* values of the models are marked bold). Predictor variables (FE, FE + SE) and outcome variables (HSI and FLI) were log-transformed

Model A (crude model) adjusted for sex and age at blood withdrawal

Model B additionally adjusted for: maternal high educational status (yes/no), smokers in household (yes/no), FMI in adolescence (kg/m^2^)

*FE* fructose excretion, *FE + SE* fructose + sucrose excretion, *HIS* hepatic steatosis index, *FLI* fatty liver index

### Associations between adolescent dietary fructose intake and adult fatty liver indices

There were no significant associations of dietary fructose intake with HSI or FLI (Table 5). Among the other sugar intake variables, only FS was consistently negatively associated with both fatty liver indices. Here, higher FS intakes were associated with lower levels of HSI (model A, *P*_trend_ = 0.02; model B, *P*_trend_ = 0.04; model C, *P*_trend_ = 0.02) and FLI (model C, *P*_trend_ = 0.03).

### Association between adolescent urinary fructose excretion and adult fatty liver indices

A higher adolescent FE was related to higher FLI values in adulthood (model B, *P*_trend_ = 0.03). Similarly, adolescent FE + SE was related to adult FLI (model B, *P*_trend_ = 0.01). FE and FE + SE were both not associated with adult HSI (Table 6).

### Sensitivity analyses

All sensitivity analyses yielded results similar to those obtained with the full sample (data shown in supplementary material S1-S5).

## Discussion

In the present study, a unique database was used to provide contradictory findings regarding the role of fructose intake in adolescence for adult liver health. While urinary FE as well as FE + SE in adolescence was positively associated with FLI in adulthood, no associations were found between self-reported fructose intake in adolescence and both fatty liver indices in adulthood. In addition, self-reported FS intake was even inversely associated with adult HSI and FLI.

Most intervention studies analysed in meta-analyses, suggest an adverse effect of a high fructose intake on risk factors or markers of NAFLD (postprandial triglycerides [52], body weight [53]; intrahepatocellular lipids and ALT [54]; insulin sensitivity [6]). These observations were solely occurring in studies using very high fructose intake levels (> 25%E) or hypercaloric diets. These high intake amounts cannot be achieved by children and adolescents in Germany with their habitual diet. Even though the self-reported fructose intake in our study population was low (3.8%E), our biomarker analyses support the hypothesis that a high fructose intake—as measured by urinary FE—is associated with a higher FLI. FLI in the highest FE or FE + SE excretion tertiles were 17 and 27% higher than those in the lowest tertiles. However, the least square means of the analyses of FE + SE showed a less clear association with FLI. Since both fatty liver indices are considered equivalent as parameters for liver fat [37] and the analyses of HSI did not statistically confirm these results, our data should be interpreted cautiously.

Of note, although the used biomarkers (FE; FE + SE) reflect total fructose intake from all food sources, studies actually validate or use them as predictive biomarkers for total sugar intake [10].

There are a number of potential explanations for the observed long-term “benefits” of higher adolescent FS intakes using dietary data. Self-reported fructose intake reflects at least in part the intake of fruits, vegetables and juices. Since these food groups also contain high amounts of phytochemicals, they can also promote health by reducing oxidative stress [55]. In the Helsinki Birth Cohort Study, an inverse association between self-reported fructose intake and FLI was observed among 1611 healthy elderly people (mean age: 61 years) [56]. The authors suggested that the possible protective impact of fruit and juice intake on health may overcome a possibly harmful impact of fructose intake from other sources like sugar-sweetened beverages [56]. Fruit, vegetable and juice intake also may have affected our results. Our observation that FS intake, which increased with juice intake, was inversely associated with fatty liver indices, whereas AS intake (i.e. sugar from SSB and sweets) showed no association with fatty liver indices, supports this argument. Since, high multicollinearity between nutrients and phytochemicals from fruit, vegetables and juices impede the differentiation of effects, confounding by fructose-adjusted flavonoids or GI appeared irrelevant in our analysis.

However, an investigation of the associations between total fructose intake (the sum of fructose intake and half of sucrose intake) from individual food groups (sugar-sweetened beverages, juices, fruits and vegetables as well as sweets) and both fatty liver indices showed no significant results (see supplementary material table S6).

Apart from fruit, vegetables and juice intake, many other factors, such as anthropometric [57, 58] and lifestyle factors [57, 59, 60], are associated with fructose intake or affect liver health and therefore the fatty liver indices. We were able to consider many potential confounders in our main or in sensitivity analyses, yet residual confounding [49] cannot be precluded. The long-term associations of total lifestyle factors during adolescence, e.g. as a pattern or score, with fatty liver indices in adulthood should be explored in future analyses.

Although sensitivity analyses excluding under-reporters of energy intake [22] yielded similar results, a possible explanation for the contrasting results between the analyses using dietary intake and urinary biomarker data is selective under-reporting of sugar-rich foods, e.g. sugar-sweetened beverages or sweets. Up till now, there is no reliable statistical method to identify selective sugar under-reporting. However, the analyses of predictive biomarkers of sugar intake in the current investigation suggest an impact of selective under-reporting on our findings (Tables 5 and 6).

The main strength of the present study is the longitudinal design of the DONALD study including the long follow-up, which allows the investigation of the relationship between fructose intake in adolescence and fatty liver indices in young adulthood. In addition, our continuously updated in-house nutrient database LEBTAB [25] allows the consideration of fructose and different types of fructose-containing sugars (TS, FS, AS). Our study is able to consider brand-specific sugar content in commercial products as well as sugars or sweetening agents, such as syrups and honey, which are used for food preparation at home. Furthermore, the urine analyses were carried out in established laboratories by scientists with years of experience in the measurement of sugar excretion in 24-h urine samples, respectively.

Our study is limited by the availability of one blood sample in young adulthood only. A further limitation of the methods used in the present study is the handling of our urine samples, which were frozen without preservatives for a long time period (the earliest 24-h urine was collected in 1985). Little is known about the stability of fructose and sucrose in urine so far. Luceri et al. [61] were the first to examine urinary biomarkers for sugar intake referring only to the instability of sucrose in urine samples stored at room temperature. Since our samples were stored at less than − 12 °C during the collection period at home as well as at − 22 °C in study institute, our samples remained frozen until use. The generalizability of our results is limited due to the relatively high SES of the DONALD study population [23] and high SES is known to correlate with lower dietary sugar intake [62]. Nevertheless, our sugar intake data are similar to sugar intake in representative German nutrition surveys [20, 63] as well as our sugar excretion data are similar to sugar excretion in other study populations [64–66].

In conclusion, the observed associations of our analyses using DONALD study data are inconsistent. We observed no association between self-reported fructose intake and fatty liver indices. The unexpected results regarding self-reported sugar intake (FS and TS) and fatty liver indices can be due to the potential healthy impact of fruit and juice intake, which have been shown to be an important source of dietary sugar among children and adolescents in Germany [19, 67, 68]. Although the weighed dietary records used in the DONALD study are considered to be a reliable and valid dietary survey method, another explanation for the observed inverse association between self-reported sugar intake and fatty liver indices is selective underreporting of high-sugar foods. This assumption is supported by the results of the biomarker analyses, which corresponded to the hypothesis of a high-dietary sugar intake, in particular fructose, as a risk factor for NAFLD. However, it is not clear whether FE as well as FE + SE serve as biomarkers for total sugar, free sugar or even fructose. Hence, further examinations estimating exposure by means of urinary excretion as well as dietary intake levels appear warranted.

## Supplementary Information

Below is the link to the electronic supplementary material.

Supplementary file1 (DOCX 42 KB)

## References

[1] Younossi ZM, Koenig AB, Abdelatif D (2016). Global epidemiology of nonalcoholic fatty liver disease-Meta-analytic assessment of prevalence, incidence, and outcomes. Hepatology.

[2] Roden M (2006). Mechanisms of Disease: hepatic steatosis in type 2 diabetes–pathogenesis and clinical relevance. Nat Clin Pract Endocrinol Metab.

[3] Tilg H, Moschen AR, Roden M (2017). NAFLD and diabetes mellitus. Nat Rev Gastroenterol Hepatol.

[4] Stanhope KL, Goran MI, Bosy-Westphal A (2018). Pathways and mechanisms linking dietary components to cardiometabolic disease: thinking beyond calories. Obes Rev.

[5] Softic S, Stanhope KL, Boucher J (2020). Fructose and hepatic insulin resistance. Crit Rev Clin Lab Sci.

[6] Ter Horst KW, Schene MR, Holman R (2016). Effect of fructose consumption on insulin sensitivity in nondiabetic subjects: a systematic review and meta-analysis of diet-intervention trials. Am J Clin Nutr.

[7] Jensen T, Abdelmalek MF, Sullivan S (2018). Fructose and sugar: a major mediator of non-alcoholic fatty liver disease. J Hepatol.

[8] Johnston RD, Stephenson MC, Crossland H (2013). No difference between high-fructose and high-glucose diets on liver triacylglycerol or biochemistry in healthy overweight men. Gastroenterology.

[9] Schwimmer JB, Ugalde-Nicalo P, Welsh JA (2019). Effect of a low free sugar diet vs usual diet on nonalcoholic fatty liver disease in adolescent boys: a randomized clinical trial. JAMA.

[10] Tasevska N (2015). Urinary sugars–a biomarker of total sugars intake. Nutrients.

[11] Poppitt SD, Swann D, Black AE (1998). Assessment of selective under-reporting of food intake by both obese and non-obese women in a metabolic facility. Int J Obes Relat Metab Disord.

[12] Johner SA, Libuda L, Shi L (2010). Urinary fructose: a potential biomarker for dietary fructose intake in children. Eur J Clin Nutr.

[13] MENZIES ISAN (1974). Absorption of Intact Oligosaccharide in Health and Disease. Biochem Soc Trans.

[14] Dietz WH (1994). Critical periods in childhood for the development of obesity. Am J Clin Nutr.

[15] Buyken AE, Mitchell P, Ceriello A (2010). Optimal dietary approaches for prevention of type 2 diabetes: a life-course perspective. Diabetologia.

[16] Vos MB, Kimmons JE, Gillespie C (2008). Dietary fructose consumption among US children and adults: the third national health and nutrition examination survey. Medscape J Med.

[17] Sluik D, Engelen AI, Feskens EJ (2015). Fructose consumption in the Netherlands: the Dutch National Food Consumption Survey 2007–2010. Eur J Clin Nutr.

[18] Perrar I, Schmitting S, Della Corte KW (2020). Age and time trends in sugar intake among children and adolescents: results from the DONALD study. Eur J Nutr.

[19] Perrar I, Schadow AM, Schmitting S (2019). Time and age trends in free sugar intake from food groups among children and adolescents between 1985 and 2016. Nutrients.

[20] Heuer T (2018). Zuckerkonsum in Deutschland. Aktuel Ernahrungsmed.

[21] Kersting M, Sichert-Hellert W, Lausen B (1998). Energy intake of 1 to 18 year old German children and adolescents. Z Ernahrungswiss.

[22] Sichert-Hellert W, Kersting M, Schöch G (1998). Underreporting of energy intake in 1 to 18 year old German children and adolescents. Z Ernahrungswiss.

[23] Kroke A, Manz F, Kersting M (2004). The DONALD Study. History, current status and future perspectives. Eur J Nutr.

[24] Buyken AE, Alexy U, Kersting M (2012). Die DONALD Kohorte. Ein aktueller Überblick zu 25 Jahren Forschung im Rahmen der Dortmund Nutritional and Anthropometric Longitudinally Designed Study (The DONALD cohort. An updated overview on 25 years of research based on the Dortmund Nutritional and Anthropometric Longitudinally Designed study). Bundesgesundheitsblatt Gesundheitsforschung Gesundheitsschutz.

[25] Sichert-Hellert W, Kersting M, Chahda C (2007). German food composition database for dietary evaluations in children and adolescents. J Food Compos Anal.

[26] Cummings JH, Stephen AM (2007). Carbohydrate terminology and classification. Eur J Clin Nutr.

[27] Swan GE, Powell NA, Knowles BL (2018). A definition of free sugars for the UK. Public Health Nutr.

[28] Slaughter MH, Lohman TG, Boileau RA (1988). Skinfold equations for estimation of body fatness in children and youth. Hum Biol.

[29] Durnin JV, Womersley J (1974). Body fat assessed from total body density and its estimation from skinfold thickness: measurements on 481 men and women aged from 16 to 72 years. Br J Nutr.

[30] Du Bois D, Du Bois EF (1916). A formula to estimate the approximate surface area if height and weight be known. Arch Intern Medicine.

[31] Buyken AE, Karaolis-Danckert N, Remer T (2009). Association of prepubertal body composition in healthy girls and boys with the timing of early and late pubertal markers. Am J Clin Nutr.

[32] Preece MA, Baines MJ (1978). A new family of mathematical models describing the human growth curve. Ann Hum Biol.

[33] Bartels H, Cikes M (1969). Über chromogene der kreatininbestimmung nach Jaffé. Clin Chim Acta.

[34] Fawcett JK, Scott JE (1960). A rapid and precise method for the determination of urea. J Clin Pathol.

[35] Patton CJ, Crouch SR (1977). Spectrophotometric and kinetics investigation of the Berthelot reaction for the determination of ammonia. Anal Chem.

[36] Hatziagelaki E, Herder C, Tsiavou A (2015). Serum chemerin concentrations associate with beta-cell function, but not with insulin resistance in individuals with non-alcoholic fatty liver disease (NAFLD). PLoS ONE.

[37] Kahl S, Straßburger K, Nowotny B (2014). Comparison of liver fat indices for the diagnosis of hepatic steatosis and insulin resistance. PLoS ONE.

[38] European Association for the Study of the Liver (EASL), European Association for the Study of Diabetes (EASD), European Association for the Study of Obesity (EASO) (2016) EASL-EASD-EASO Clinical Practice Guidelines for the management of non-alcoholic fatty liver disease. J Hepatol 64:1388–1402. 10.1016/j.jhep.2015.11.004 10.1016/j.jhep.2015.11.004 27062661

[39] Lee J-H, Kim D, Kim HJ (2010). Hepatic steatosis index: a simple screening tool reflecting nonalcoholic fatty liver disease. Dig Liver Dis.

[40] Bedogni G, Bellentani S, Miglioli L (2006). The Fatty Liver Index: a simple and accurate predictor of hepatic steatosis in the general population. BMC Gastroenterol.

[41] Booth ML, Okely AD, Chey TN (2002). The reliability and validity of the adolescent physical activity recall questionnaire. Med Sci Sports Exerc.

[42] Opper E, Worth A, Wagner M (2007). Motorik-Modul (MoMo) im Rahmen des Kinder- und Jugendgesundheitssurveys (KiGGS). Motorische Leistungsfähigkeit und körperlich-sportliche Aktivität von Kindern und Jugendlichen in Deutschland (The module "Motorik" in the German Health Interview and Examination Survey for Children and Adolescents (KiGGS). Motor fitness and physical activity of children and young people). Bundesgesundheitsblatt Gesundheitsforschung Gesundheitsschutz.

[43] Tasevska N, Runswick SA, McTaggart A (2005). Urinary sucrose and fructose as biomarkers for sugar consumption. Cancer Epidemiol Biomarkers Prev.

[44] Willett WC, Howe GR, Kushi LH (1997). Adjustment for total energy intake in epidemiologic studies. Am J Clin Nutr.

[45] Taylor TP, Wang W, Shrayyef MZ (2006). Glomerular filtration rate can be accurately predicted using lean mass measured by dual-energy X-ray absorptiometry. Nephrol Dial Transplant.

[46] Victora CG, Huttly SR, Fuchs SC (1997). The role of conceptual frameworks in epidemiological analysis: a hierarchical approach. Int J Epidemiol.

[47] Maldonado G, Greenland S (1993). Simulation study of confounder-selection strategies. Am J Epidemiol.

[48] Kirkwood BR (2003). SJAC essential medical statistics.

[49] Tasevska N, Midthune D, Potischman N (2011). Use of the predictive sugars biomarker to evaluate self-reported total sugars intake in the Observing Protein and Energy Nutrition (OPEN) study. Cancer Epidemiol Biomarkers Prev.

[50] Schofield WN (1985). Predicting basal metabolic rate, new standards and review of previous work. Hum Nutr Clin Nutr.

[51] Livingstone MB, Robson PJ (2000). Measurement of dietary intake in children. Proc Nutr Soc.

[52] David Wang D, Sievenpiper JL, de Souza RJ (2014). Effect of fructose on postprandial triglycerides: a systematic review and meta-analysis of controlled feeding trials. Atherosclerosis.

[53] Sievenpiper JL, de Souza RJ, Mirrahimi A (2012). Effect of fructose on body weight in controlled feeding trials: a systematic review and meta-analysis. Ann Intern Med.

[54] Chiu S, Sievenpiper JL, de Souza RJ (2014). Effect of fructose on markers of non-alcoholic fatty liver disease (NAFLD): a systematic review and meta-analysis of controlled feeding trials. Eur J Clin Nutr.

[55] Tonin FS, Steimbach LM, Wiens A (2015). Impact of natural juice consumption on plasma antioxidant status: a systematic review and meta-analysis. Molecules.

[56] Kanerva N, Sandboge S, Kaartinen NE (2014). Higher fructose intake is inversely associated with risk of nonalcoholic fatty liver disease in older Finnish adults. Am J Clin Nutr.

[57] Chalasani N, Younossi Z, Lavine JE (2012). The diagnosis and management of non-alcoholic fatty liver disease: practice Guideline by the American Association for the Study of Liver Diseases, American College of Gastroenterology, and the American Gastroenterological Association. Hepatology.

[58] Pollock NK, Bundy V, Kanto W (2012). Greater fructose consumption is associated with cardiometabolic risk markers and visceral adiposity in adolescents. J Nutr.

[59] Weitzman M, Cook S, Auinger P (2005). Tobacco smoke exposure is associated with the metabolic syndrome in adolescents. Circulation.

[60] Terry-McElrath YM, OʼMalley PM, Johnston LD,  (2014). Energy drinks, soft drinks, and substance use among United States secondary school students. J Addict Med.

[61] Luceri C, Caderni G, Lodovici M (1996). Urinary excretion of sucrose and fructose as a predictor of sucrose intake in dietary intervention studies. Cancer Epidemiol Biomarkers Prev.

[62] Thompson FE, McNeel TS, Dowling EC (2009). Interrelationships of added sugars intake, socioeconomic status, and race/ethnicity in adults in the United States: National Health Interview Survey 2005 (ADAJ-D-08-00562R1). J Am Diet Assoc.

[63] Mensink GB (2007) Die aktuelle Nährstoffversorgung von Kindern und Jugendlichen in Deutschland. Ernährungsumschau 54:636–646

[64] Intemann T, Pigeot I, de Henauw S (2019). Urinary sucrose and fructose to validate self-reported sugar intake in children and adolescents: results from the I.Family study. Eur J Nutr.

[65] Joosen AMCP, Kuhnle GGC, Runswick SA (2008). Urinary sucrose and fructose as biomarkers of sugar consumption: comparison of normal weight and obese volunteers. Int J Obes (Lond).

[66] Kuhnle GGC, Joosen AMCP, Wood TR (2008). Detection and quantification of sucrose as dietary biomarker using gas chromatography and liquid chromatography with mass spectrometry. Rapid Commun Mass Spectrom.

[67] Mensink GBM, Kleiser C, Richter A (2007). Lebensmittelverzehr bei Kindern und Jugendlichen in Deutschland. Ergebnisse des Kinder- und Jugendgesundheitssurveys (KiGGS) (Food consumption of children and adolescents in Germany. Results of the German Health Interview and Examination Survey for Children and Adolescents (KiGGS)). Bundesgesundheitsblatt Gesundheitsforschung Gesundheitsschutz.

[68] Rabenberg M, Mensink GBM (2013) Limo, Saft & Co - Konsum zuckerhaltiger Getränke in Deutschland. Hrsg. Robert Koch-Institut Berlin. GBE kompakt 4(1). 10.25646/3036

