# Supplementary material for: Relevance of fructose intake in adolescence for fatty liver indices in young adulthood
Source: Eur J Nutr. 2021 Jan 19;60(6):3029–41. doi: 10.1007/s00394-020-02463-2 (PMC8354997; doi:10.1007/s00394-020-02463-2)
Supplement: Supplementary file 1 — Supplementary file1 (DOCX 42 KB) [file 394_2020_2463_MOESM1_ESM.docx]

**Table S1:** Sensitivity analyses: Relation of fructose intake as well as total, free and added sugar intake in adolescence (males: 9.5-16.5 years; females: 8.5-15.5 years) with indices of non-alcoholic fatty liver diseases (hepatic steatosis index (HSI), fatty liver index (FLI)) in young adulthood (18-36 years) without underreported records (1415 records from 245 DONALD participants)

|  | **Predicted means for HSI in adulthood in tertiles of sugar intake in adolescence** | | |  | **Predicted means for FLI in adulthood in tertiles of sugar intake in adolescence** | | |  |
| --- | --- | --- | --- | --- | --- | --- | --- | --- |
|  | **Low**  **intake**  **(T1)** | **Moderate**  **intake**  **(T2)** | **High**  **intake**  **(T3)** | **P_trend_** | **Low**  **intake**  **(T1)** | **Moderate**  **intake**  **(T2)** | **High**  **intake**  **(T3)** | **P_trend_** |
| **Fructose intake** |  |  |  |  |  |  |  |  |
| Model A | 31.5 (30.5; 32.6) | 30.6 (29.6; 31.7) | 30.2 (26.2; 31.2) | 0.07 | 11.2 (9.1; 13.7) | 9.4 (7.7; 11.5) | 8.9 (7.3; 10.9) | 0.29 |
| Model B | 31.3 (30.4; 32.3) | 31.2 (30.2; 32.1) | 30.2 (29.3; 31.2) | 0.13 | 10.7 (8.9; 12.9) | 10.3 (8.6; 12.4) | 9.0 (7.5; 10.9) | 0.49 |
| Model C | 31.3 (30.4; 32.3) | 31.2 (30.2; 32.1) | 30.2 (29.3; 31.2) | 0.11 | 11.2 (9.3; 13.5) | 10.5 (8.7; 12.6) | 8.4 (6.9; 10.2) | 0.19 |
| **Total sugar intake** |  |  |  |  |  |  |  |  |
| Model A | 32.0 (30.9; 33.1) | 30.3 (29.3; 31.4) | 30.1 (29.1; 31.1) | 0.06 | 11.8 (9.6; 14.4) | 8.7 (7.1; 10.6) | 9.2 (7.6; 11.3) | 0.35 |
| Model B | 32.0 (31.0; 33.0) | 30.5 (29.6; 31.4) | 30.3 (29.4; 31.3) | 0.03 | 11.7 (9.7; 14.1) | 8.9 (7.4; 10.7) | 9.7 (8.1; 11.7) | 0.29 |
| Model C | 32.1 (31.1; 33.2) | 30.5 (29.6; 31.5) | 30.1 (29.2; 31.1) | **0.01** | 12.5 (10.3; 15.1) | 9.1 (7.6; 10.8) | 8.9 (7.3; 10.8) | 0.05 |
| **Free Sugar intake** |  |  |  |  |  |  |  |  |
| Model A | 32.3 (31.3; 33.5) | 29.8 (28.8; 30.8) | 30.3 (29.3; 31.3) | 0.05 | 13.0 (10.7;15.9) | 7.7 (6.3; 9.4) | 9.4 (7.7; 11.5) | 0.18 |
| Model B | 32.1 (31.0; 33.1) | 30.3 (29.4; 31.2) | 30.5 (29.6; 31.4) | 0.03 | 12.4 (10.3; 14.9) | 8.4 (7.0; 10.1) | 9.7 (8.1; 11.7) | 0.17 |
| Model C | 32.2 (31.2; 33.2) | 30.3 (29.4; 31.2) | 30.3 (29.4; 31.3) | **0.02** | 13.0 (10.8; 15.7) | 8.5 (7.1; 10.2) | 9.0 (7.5; 10.9) | **0.03** |
| **Added Sugar intake** |  |  |  |  |  |  |  |  |
| Model A | 31.6 (30.6; 32.7) | 29.9 (28.9; 30.9) | 30.8 (29.8; 31.9) | 0.68 | 11.1 (9.1; 13.6) | 8.3 (6.8; 10.2) | 10.2 (8.3;12.4) | 0.96 |
| Model B | 31.6 (30.6; 32.6) | 30.5 (29.6; 31.4) | 30.7 (29.8; 31.7) | 0.24 | 10.8 (8.9; 13.2) | 9.3 (7.7; 11.2) | 10.1 (8.4;12.1) | 0.54 |
| Model C | 31.6 (30.6; 32.6) | 30.5 (29.6; 31.4) | 30.7 (29.8; 31.7) | 0.23 | 11.0 (9.0; 13.3) | 9.4 (7.8; 11.4) | 9.8 (8.1; 11.7) | 0.34 |

Values are least square means (95% confidence interval) of HSI/FLI in the respective sex-specific tertiles of sugar intake

HSI= hepatic steatosis index; FLI=fatty liver index;

P-values for models are based on linear multivariable regression analyses. Outcome variables (HSI and FLI) were log transformed.

Model A (crude model) adjusted for sex and age at blood withdrawal

Model B = Model A additionally adjusted for: gestational weight gain (kg), maternal high educational status (yes/no), maternal overweight (yes/no), FMI in adolescence (kg/m2)

Model C = Model B additionally adjusted for energy-yielding nutrients (adolescent saturated fatty acids intake (residuals))

**Table S2:** Sensitivity analyses: Relation of kalibrated fructose excretion (FE) as well as kalibrated fructose+sucrose (FE+SE) excretion in adolescence (males: 9.5-16.5 years; females: 8.5-15.5 years) with indices of non-alcoholic fatty liver diseases (hepatic steatosis index (HSI), fatty liver index (FLI)) in young adulthood (18-36 years) (n=223)

|  | **Predicted means for HSI in adulthood in tertiles of sugar excretion in adolescence** | | |  | **Predicted means for FLI in adulthood in tertiles of sugar excretion in adolescence** | | |  |
| --- | --- | --- | --- | --- | --- | --- | --- | --- |
|  | **Low**  **excretion**  **(T1)** | **Moderate**  **excretion**  **(T2)** | **High**  **excretion**  **(T3)** | **P_trend_** | **Low**  **excretion**  **(T1)** | **Moderate**  **excretion**  **(T2)** | **High**  **excretion**  **(T3)** | **P_trend_** |
| **FE** |  |  |  |  |  |  |  |  |
| Model A | 30.8 (29.7; 31.8) | 30.4 (29.4; 31.5) | 30.4 (29.4; 31.5) | 0.45 | 9.4 (7.7; 11.5) | 9.4 (7.7; 11.6) | 9.5 (7.8; 11.7) | 0.30 |
| Model B | 30.3 (29.4; 31.3) | 30.4 (29.5; 31.4) | 30.8 (29.9; 31.8) | 0.05 | 8.4 (6.9; 10.1) | 9.3 (7.8; 11.2) | 10.1 (8.4; 12.2) | **0.03** |
| **FE+SE** |  |  |  |  |  |  |  |  |
| Model A | 30.8 (29.7; 31.8) | 30.3 (29.2; 31.3) | 30.6 (29.6; 31.7) | 0.81 | 9.6 (7.8; 11.8) | 8.9 (7.3; 10.9) | 9.9 (8.1; 12.1) | 0.31 |
| Model B | 30.3 (29.3; 31.3) | 30.1 (29.2; 31.0) | 31.2 (30.3; 32.3) | 0.18 | 8.5 (7.0; 10.3) | 8.5 (7.1; 10.2) | 11.0 (9.1; 13.2) | **0.03** |

Values are least square means (95% confidence interval) of HSI/FLI in respective sex-specific tertiles of sugar excretion

FE= fructose excretion; FE+SE=fructose+sucrose excretion; HSI= hepatic steatosis index; FLI=fatty liver index; %E=percentage of total energy intake

P-values for models are based on linear multivariable regression analyses. Predictor variables (FE, FE+SE) and outcome variables (HSI and FLI) were log transformed.

Model A (crude model) adjusted for sex and age at blood withdrawal

Model B additionally adjusted for: maternal high educational status (yes/no), smokers in household (yes/no), FMI in adolescence (kg/m^2^)

**Table S3:** Sensitivity analyses for adult smoking status: Relation of fructose intake as well as total, free and added sugar intake in adolescence (males: 9.5-16.5 years; females: 8.5-15.5 years) with indices of non-alcoholic fatty liver diseases (hepatic steatosis index (HSI), fatty liver index (FLI)) in young adulthood (18-36 years) (n=224)

|  | **Predicted means for HSI in adulthood in tertiles of sugar intake in adolescence** | | |  | **Predicted means for FLI in adulthood in tertiles of sugar intake in adolescence** | | |  |
| --- | --- | --- | --- | --- | --- | --- | --- | --- |
|  | **Low**  **intake**  **(T1)** | **Moderate**  **intake**  **(T2)** | **High**  **intake**  **(T3)** | **P_trend_** | **Low**  **intake**  **(T1)** | **Moderate**  **intake**  **(T2)** | **High**  **intake**  **(T3)** | **P_trend_** |
| **Fructose intake** |  |  |  |  |  |  |  |  |
| Model A | 31.5 (30.4; 32.6) | 30.3 (29.3; 31.4) | 30.1 (29.0; 31.1) | 0.10 | 10.9 (8.9; 13.4) | 9.4 (7.7; 11.6) | 8.5 (6.9; 10.4) | 0.38 |
| Model B | 31.2 (30.2; 32.2) | 30.7 (29.7 31.7) | 30.3 (29.3; 31.3) | 0.16 | 10.4 (8.5; 12.6) | 10.0 (8.3; 12.2) | 8.8 (7.3; 10.8) | 0.57 |
| Model C  Conditional Model | 31.3 (30.3; 32.4)  31.4 (30.4; 32.5) | 30.7 (29.7; 31.7)  30.9 (29.9; 31.9) | 30.2 (29.2; 31.2)  30.5 (29.4; 31.6) | 0.09  0.16 | 10.9 (8.9; 13.3)  11.3 (9.2; 13.8) | 10.0 (8.3; 12.1)  10.6 (8.7; 13.0) | 8.4 (6.8; 10.3)  9.1 (7.4; 11.3) | 0.18  0.37 |
| **Total sugar intake** |  |  |  |  |  |  |  |  |
| Model A | 32.1 (31.1; 33.2) | 30.0 (29.0; 31.0) | 29.8 (28.8; 30.8) | **0.03** | 11.9 (9.7; 14.7) | 8.2 (6.7;10.1) | 8.9 (7.2; 10.9) | 0.20 |
| Model B | 32.2 (31.2; 33.2) | 29.9 (29.0; 30.8) | 30.2 (29.3; 31.2) | 0.05 | 12.0 (9.9; 14.6) | 8.1 (6.8; 9.8) | 9.6 (7.9; 11.7) | 0.35 |
| Model C  Conditional Model | 32.5 (31.4; 33.5)  32.7 (31.6; 33.8) | 30.0 (29.1; 30.9)  30.2 (29.3; 31.1) | 29.9 (28.9; 30.9)  30.2 (29.1; 31.2) | **0.02**  **0.03** | 12.9 (10.6; 15.7)  13.7 (11.1; 16.8) | 8.2 (6.8; 9.9)  8.8 (7.3; 10.7) | 8.8 (7.1; 10.7)  9.5 (7.7; 11.7) | 0.06  0.10 |
| **Free Sugar intake** |  |  |  |  |  |  |  |  |
| Model A | 32.4 (31.3; 33.5) | 29.6 (28.6; 30.5) | 30.1 (29.1; 31.1) | **0.01** | 12.9 (10.5; 15.8) | 7.8 (6.4; 9.5) | 8.8 (7.2; 10.8) | 0.05 |
| Model B | 32.3 (31.3; 33.3) | 29.7 (28.8; 30.6) | 30.4 (29.4; 31.3) | **0.02** | 12.6 (10.4; 15.3) | 8.0 (6.6; 9.6) | 9.3 (7.7; 11.3) | 0.11 |
| Model C  Conditional Model | 32.5 (31.4; 33.5)  32.7 (31.6; 33.8) | 29.7 (28.9; 30.7)  30.0 (29.0; 30.9) | 30.2 (29.2; 31.2)  30.4 (29.4; 31.5) | **0.01**  **0.01** | 13.3 (11.0; 16.2)  14.3 (11.7; 17.6) | 8.1 (6.7; 9.7)  8.6 (7.1; 10.4) | 8.6 (7.0; 10.5)  9.3 (7.6; 11.4) | **0.01**  **0.02** |
| **Added Sugar intake** |  |  |  |  |  |  |  |  |
| Model A | 31.6 (30.5; 32.7) | 30.0 (29.0; 31.0) | 30.3 (29.3; 31.4) | 0.32 | 11.4 (9.3; 14.0) | 8.2 (6.7; 10.0) | 9.4 (7.7; 11.6) | 0.50 |
| Model B | 31.8 (30.7; 32.8) | 30.2 (29.3; 31.0) | 30.4 (29.5; 31.4) | 0.23 | 11.4 (9.3; 13.8) | 8.3 (6.9; 10.0) | 9.4 (7.7; 11.4) | 0.46 |
| Model C | 31.8 (30.8; 32.9) | 30.2 (29.3; 31.2) | 30.3 (29.4; 31.3) | 0.19 | 11.6 (9.5; 14.1) | 8.5 (7.0; 10.2) | 9.1 (7.4; 11.0) | 0.26 |
| Conditional Model | 32.0 (30.9; 33.1) | 30.4 (29.5; 31.4) | 30.5 (29.5; 31.6) | 0.17 | 12.6 (10.2; 15.4) | 9.3 (7.6; 11.3) | 9.8 (8.0; 12.0) | 0.23 |

Values are least square means (95% confidence interval) of HSI/FLI in the respective sex-specific tertiles of sugar intake

HSI= hepatic steatosis index; FLI=fatty liver index; SSB=sugar sweetened beverages

P-values for models are based on linear multivariable regression analyses. Outcome variables (HSI and FLI) were log transformed.

Model A (crude model) adjusted for sex and age at blood withdrawal

Model B = Model A additionally adjusted for: gestational weight gain (kg), maternal high educational status (yes/no), maternal overweight (yes/no), FMI in adolescence (kg/m2)

Model C = Model B additionally adjusted for energy-yielding nutrients (adolescent saturated fatty acids intake (residuals))

Conditional Model = Model C additionally adjusted for adult smoking status (yes/no)

**Table S4:** Sensitivity analyses for adult smoking status: Relation of kalibrated fructose excretion (FE) as well as kalibrated fructose+sucrose (FE+SE) excretion in adolescence (males: 9.5-16.5 years; females: 8.5-15.5 years) with indice of non-alcoholic fatty liver diseases (hepatic steatosis index (HSI)) in young adulthood (18-36 years) (n=223)

|  | **Predicted means for HSI in adulthood in tertiles of sugar excretion in adolescence** | | |  |
| --- | --- | --- | --- | --- |
|  | **Low**  **excretion**  **(T1)** | **Moderate**  **excretion**  **(T2)** | **High**  **excretion**  **(T3)** | **P_trend_** |
| **FE** |  |  |  |  |
| Model A | 30.6 (29.5; 31.7) | 30.03 (29.3; 31.4) | 30.3 (29.3; 31.4) | 0.42 |
| Model B | 30.3 (29.3; 31.3) | 30.4 (29.5; 31.4) | 30.7 (29.7; 31.8) | 0.07 |
| Conditional Model | 30.2 (29.2; 31.2) | 30.4 (29.5; 31.4) | 30.8 (29.8; 31.8) | **0.04** |
| **FE+SE** |  |  |  |  |
| Model A | 30.6 (29.6; 31.7) | 30.1 (29.0; 31.1) | 30.6 (29.5; 31.7) | 0.65 |
| Model B | 30.4 (29.4; 31.4) | 29.9 (28.9; 30.8) | 31.2 (30.2; 32.3) | 0.18 |
| Conditional Model | 30.3 (29.4; 31.3) | 29.9 (28.9; 30.8) | 31.2 (30.2; 32.3) | 0.14 |

Values are least square means (95% confidence interval) of HSI/FLI in respective sex-specific tertiles of sugar excretion

FE= fructose excretion; FE+SE=fructose+sucrose excretion; HSI= hepatic steatosis index; FLI=fatty liver index; %E=percentage of total energy intake

P-values for models are based on linear multivariable regression analyses. Predictor variables (FE, FE+SE) and outcome variables (HSI and FLI) were log transformed.

Model A (crude model) adjusted for sex and age at blood withdrawal

Model B additionally adjusted for: maternal high educational status (yes/no), smokers in household (yes/no), FMI in adolescence (kg/m^2^)

**Table S5:** Sensitivity analyses for levels of adult physical activity: Relation of fructose intake as well as total, free and added sugar intake in adolescence (males: 9.5-16.5 years; females: 8.5-15.5 years) with indice of non-alcoholic fatty liver diseases (fatty liver index (FLI)) in young adulthood (18-36 years) (n=169)

|  | **Predicted means for FLI in adulthood in tertiles of sugar intake in adolescence** | | |  |
| --- | --- | --- | --- | --- |
|  | **Low**  **intake**  **(T1)** | **Moderate**  **intake**  **(T2)** | **High**  **intake**  **(T3)** | **P_trend_** |
| **Fructose intake** |  |  |  |  |
| Model A | 9.4 (7.3; 12.0) | 8.6 (6.7; 11.0) | 8.7 (6.7; 11.1) | 0.79 |
| Model B | 9.3 (7.4; 11.8) | 9.4 (7.4; 11.9) | 8.6 (6.9; 10.9) | 0.60 |
| Model C  Conditional Model | 10.3 (8.2; 13.1)  10.4 (8.2; 13.1) | 9.1 (7.3; 11.5)  9.2 (7.3; 11.6) | 7.8 (6.1; 9.9)  7.7 (6.0; 9.8) | 0.08  0.08 |
| **Total sugar intake** |  |  |  |  |
| Model A | 9.8 (7.6; 12.6) | 9.0 (7.0; 11.5) | 7.9 (6.2; 10.1) | 0.26 |
| Model B | 9.7 (7.7; 12.3) | 9.1 (7.3; 11.5) | 8.6 (6.8; 10.8) | 0.30 |
| Model C  Conditional Model | 11.0 (8.6; 14.0)  11.1 (8.7; 14.2) | 9.2 (7.3; 11.5)  9.3 (7.4; 11.6) | 7.5 (5.9; 9.5)  7.3 (5.7; 9.3) | **0.02**  **0.01** |
| **Free Sugar intake** |  |  |  |  |
| Model A | 11.6 (9.1; 14.8) | 7.4 (5.8; 9.5) | 8.2 (6.4; 10.4) | 0.10 |
| Model B | 11.6 (9.2; 14.7) | 7.6 (6.1; 9.5) | 8.7 (7.0; 11.0) | 0.23 |
| Model C  Conditional Model | 12.2 (9.7; 15.4)  12.5 (9.9; 15.7) | 7.8 (6.3; 9.8)  7.9 (6.3; 9.9) | 8.0 (6.3; 9.8)  7.8 (6.2; 9.8) | **0.03**  **0.01** |
| **Added Sugar intake** |  |  |  |  |
| Model A | 10.9 (8.5; 14.0) | 7.4 (5.8; 9.4) | 8.7 (6.8; 11.1) | 0.41 |
| Model B | 11.0 (8.6; 14.1) | 7.9 (6.3; 10.0) | 9.0 (7.1; 11.3) | 0.60 |
| Model C | 11.1 (8.7; 14.2) | 8.2 (6.5; 10.2) | 8.6 (6.8;10.8) | 0.38 |
| Conditional Model | 11.2 (8.8; 14.4) | 8.3 (6.6; 10.4) | 8.4 (6.7; 10.6) | 0.27 |

Values are least square means (95% confidence interval) ofFLI in the respective sex-specific tertiles of sugar intake

FLI=fatty liver index; SSB=sugar sweetened beverages

P-values for models are based on linear multivariable regression analyses. Outcome variables (HSI and FLI) were log transformed.

Model A (crude model) adjusted for sex and age at blood withdrawal

Model B = Model A additionally adjusted for: gestational weight gain (kg), maternal high educational status (yes/no), maternal overweight (yes/no), FMI in adolescence (kg/m2)

Model C = Model B additionally adjusted for energy-yielding nutrients (adolescent saturated fatty acids intake (residuals))

Conditional Model = Model C additionally adjusted for levels of adult physical activity (low/medium/high)

**Table S6:** Relation of total fructose intake from food groups (sugar sweetened bevegares (SSB), juices, fruits & vegetables, sweets) in adolescence (males: 9.5-16.5 years; females: 8.5-15.5 years) with indices of non-alcoholic fatty liver diseases (hepatic steatosis index (HSI), fatty liver index (FLI)) in young adulthood (18-36 years) (n=246)

|  | **Predicted means for HSI in adulthood in tertiles of sugar intake in adolescence** | | |  | **Predicted means for FLI in adulthood in tertiles of sugar intake in adolescence** | | |  |
| --- | --- | --- | --- | --- | --- | --- | --- | --- |
|  | **Low**  **intake**  **(T1)** | **Moderate**  **intake**  **(T2)** | **High**  **intake**  **(T3)** | **P_trend_** | **Low**  **intake**  **(T1)** | **Moderate**  **intake**  **(T2)** | **High**  **intake**  **(T3)** | **P_trend_** |
| **Fructose intake from SSB** |  |  |  |  |  |  |  |  |
| Model A | 30.8 (29.7; 31.8) | 31.4 (30.4; 32.5) | 30.2 (29.2; 31.2) | 0.98 | 9.7 (7.9; 11.9) | 10.7 (8.8; 13.1) | 9.1 (7.5; 11.1) | 0.70 |
| Model B | 31.0 (30.0; 32.0) | 31.6 (30.7; 32.6) | 30.1 (29.2; 31.0) | 0.69 | 10.1 (8.3; 12.2) | 11.0 (9.2; 13.2) | 9.0 (7.5; 10.9) | 0.87 |
| Model C | 31.0 (30.1; 32.0) | 31.6 (30.7; 32.6) | 30.0 (29.1; 31.0) | 0.62 | 10.3 (8.5; 12.4) | 11.1 (9.3; 13.3) | 8.8 (7.3; 10.6) | 0.84 |
| **Fructose intake from juices** |  |  |  |  |  |  |  |  |
| Model A | 31.2 (30.1; 32.3) | 30.9 (29.8; 31.9) | 30.3 (29.3; 31.4) | 0.04 | 10.3 (8.3; 12.7) | 10.3 (8.4; 12.6) | 9.0 (7.3; 11.0) | 0.10 |
| Model B | 30.9 (30.0; 32.0) | 31.0 (30.1; 32.0) | 30.7 (29.7; 31.7) | 0.14 | 9.9 (8.1; 12.0) | 10.6 (8.8; 12.7) | 9.6 (7.9; 12.7) | 0.28 |
| Model C | 31.0 (30.0; 32.0) | 31.0 (30.1; 32.0) | 30.7 (29.7; 31.7) | 0.10 | 10.3 (8.4; 12.5) | 10.5 (8.8; 12.6) | 9.2 (7.6; 11.2) | 0.09 |
| **Fructose intake from fruits & vegetables** |  |  |  |  |  |  |  |  |
| Model A | 30.5 (29.5; 31.6) | 31.0 (30.0; 32.1) | 30.8 (29.7; 31.8) | 0.51 | 9.1 (7.4; 11.1) | 9.9 (8.1; 12.1) | 10.5 (8.6; 12.9) | 0.67 |
| Model B | 30.8 (29.9; 31.8) | 31.0 (30.0; 31.9) | 30.9 (30.0; 31.9) | 0.43 | 9.6 (8.0; 11.6) | 9.7 (8.1; 11.6) | 10.8 (9.0; 13.0) | 0.66 |
| Model C | 30.8 (29.9; 31.8) | 31.0 (30.0; 31.9) | 30.9 (29.9; 31.9) | 0.36 | 9.9 (8.2; 11.9) | 9.7 (8.1; 11.6) | 10.5 (8.7; 12.7) | 0.99 |
| **Fructose intake from sweets** |  |  |  |  |  |  |  |  |
| Model A | 32.2 (31.1; 33.3) | 29.8 (28.8; 30.8) | 30.5 (29.5; 31.5) | 0.11 | 12.3 (10.1; 15.0) | 7.8 (6.4; 9.5) | 10.0 (8.2; 12.2) | 0.42 |
| Model B | 31.9 (31.0; 32.9) | 30.1 (29.2; 31.0) | 30.7 (29.8; 31.7) | 0.27 | 11.7 (9.8; 14.1) | 8.3 (6.9; 9.9) | 10.5 (8.7; 12.6) | 0.84 |
| Model C | 31.9 (31.0; 32.9) | 30.1 (29.2; 31.0) | 30.7 (29.8; 31.7) | 0.27 | 11.7 (9.7; 14.0) | 8.3 (6.9; 9.9) | 10.4 (8.6; 12.5) | 0.82 |

Values are least square means (95% confidence interval) of HSI/FLI in the respective sex-specific tertiles of sugar intake

HSI= hepatic steatosis index; FLI=fatty liver index; SSB=sugar sweetened beverages

P-values for models are based on linear multivariable regression analyses. Outcome variables (HSI and FLI) were log transformed.

Model A (crude model) adjusted for sex and age at blood withdrawal

Model B = Model A additionally adjusted for: gestational weight gain (kg), maternal high educational status (yes/no), maternal overweight (yes/no), FMI in adolescence (kg/m2)

Model C = Model B additionally adjusted for energy-yielding nutrients (adolescent saturated fatty acids intake (residuals))
